# Alcohol‐Induced Disinhibition of Facial Responses to Pain

**DOI:** 10.1002/ejp.70091

**Published:** 2025-08-01

**Authors:** Stefan Lautenbacher, Claudia Horn‐Hofmann, Eva Susanne Capito, Jörg Wolstein, Miriam Kunz

**Affiliations:** ^1^ Physiological Psychology Otto‐Friedrich University of Bamberg Bamberg Germany; ^2^ Medical Psychology and Sociology, Medical Faculty University of Augsburg Augsburg Germany; ^3^ Psychopathology Otto‐Friedrich University of Bamberg Bamberg Germany

## Abstract

**Background:**

Alcohol in sub‐toxic dosages has appeared to slightly reduce experimental pain in psychophysical paradigms. However, this effect may also reflect impaired scaling performance in subjective ratings. To address this, we additionally assessed facial responses as a more direct and cognitively unbiased pain measure, while acknowledging the potential confound of alcohol's effects on motor inhibitory function.

**Methods:**

We investigated 41 healthy participants (22 females) in a randomised, double‐blind, and placebo‐controlled design; targeting two moderate breath‐alcohol levels (0.6‰, 0.8‰). Before and after an alcoholic or placebo drink, painful heat stimuli were applied to the forearm. Facial responses were analysed using the Facial Action Coding System (FACS). Subjective responses were assessed using a Numerical Rating Scale (NRS). To control for alcohol's effects on motor inhibitory function, participants completed the antisaccade task, which assesses inhibitory control over reflexive motor responses (eye movement).

**Results:**

While pain ratings were unaffected, alcohol significantly affected facial responses to pain, with the high alcohol dose leading to increased facial responses. Moreover, alcohol also led to a decrease in inhibitory control, with poorer performance in the antisaccade task. Not surprisingly, we found a significant association between the alcohol‐induced increase in facial responses and the alcohol‐induced decrease in inhibitory control.

**Discussion:**

Alcohol‐induced motor disinhibition likely enhanced facial responses to pain without altering the subjective pain experience. In consequence, individuals under the influence of alcohol may facially display stronger pain levels (than experienced), which should not be interpreted as intentional exaggeration by clinicians involved in pain assessment.

**Significance Statement:**

Subtoxic doses of alcohol are known to produce weak analgesic effects. In contrast, the facial responses to pain were elevated under alcohol in the present study; probably due to an alcohol‐induced motor disinhibition. Thus, individuals under the influence of alcohol may be analgized while in parallel being facially overly pain responsive.

## 1 Introduction

Alcohol is a complex psychoactive substance whose effects on cognition and emotion vary with dose, form, route, and context of administration (Baltariu et al. 2023; Capito et al. 2017; Mintzer 2007; Schweizer and Vogel‐Sprott 2008), making its psychic impact difficult to predict. In terms of its action on pain, mainly analgesic, anaesthetic, and sedative effect components have been considered (Duarte et al. 2008; Thompson et al. 2017). There is some evidence that sub‐toxic doses of alcohol exert acute analgesic actions, that is, reducing pain sensitivity (Capito et al. 2020; Horn‐Hofmann et al. 2015, 2019; Thompson et al. 2017). However, although the consumption of alcohol is worldwide very prevalent, literature on its acute effects on pain is surprisingly scarce.

When investigating alcohol's effects on pain, it is important to consider that alcohol might not only affect pain processing but also pain reporting. Indeed, pain measures that rely on bodily memory and perceptual scaling may be altered by alcohol besides the direct effects on pain. To get a more comprehensive picture, other types of pain responses—besides self‐report—should be considered; especially those that rely less on cognitive functioning, like facial responses. Facial responses to pain are more automatic and reflex‐like and are subject to less cognitive control (Hadjistavropoulos and Craig 2002). Thus, it is interesting to see whether similar analgesic effects of sub‐toxic dosages of alcohol will be found when studying facial responses to pain.

However, given the broad effect of alcohol, it is again important to consider how alcohol might affect facial responses themselves, independently of its effect on pain. We could provide substantial evidence that facial expressions of pain are governed by a prefrontal gate of motor inhibition (Kunz et al. 2011, 2023) that exerts different levels of inhibitory control depending on the context. Thus, the intensity of facial responses seems to depend on both the intensity of the noxious event and the opening of the inhibitory motor gate for facial responses (Karmann et al. 2015, 2016). Since alcohol is known to cause motor disinhibition (Rose and Duka 2007), a pharmacological opening of the inhibitory motor gate might occur after alcohol consumption; thus, enhancing facial displays of pain.

Altogether, the study attempts to replicate the analgesic effects of subtoxic alcohol doses as assessed in similar studies using psychophysical measures; this time by quantification of facial responses to pain. Since alcohol may cause a non‐pain‐related disinhibition of facial responses, motor inhibition was assessed using the antisaccade task as a control measure. Previous findings show that performance in the antisaccade task is predictive of the degree of facial expressiveness in response to pain (Karmann et al. 2015).

We hypothesise that subtoxic doses of alcohol reduce subjective pain ratings and facial expressions of pain to a fairly similar extent. A divergence between these measures under the influence of alcohol may suggest that alcohol also induces motor disinhibition, predominantly affecting the facial responses to pain, based on a mechanism that should manifest in an impaired performance conducting the antisaccade task.

## 2 Materials and Methods

### 2.1 Participants

Participants were recruited via announcement in the local media. Individuals with severe acute or chronic illnesses, mental disorders, any conditions involving pain symptoms, alcohol use disorders, regular use of analgesics or prescription drugs (except oral contraceptives), or use of illegal substances were excluded from participation. Females had to provide a negative pregnancy test; mothers still breastfeeding were not allowed to participate. Participation in our previous study (Horn‐Hofmann et al. 2019) was another criterion for exclusion to keep subjects naïve as regards intervention. Participants were asked to refrain from smoking 1 h, from food 4 h and from alcohol and other drugs (except oral contraceptives) 24 h before attending the test sessions.

Our sample comprised a total of 41 healthy individuals (22 female; age range: 30–60 years, for mean age see Table 1), who reported drinking low to moderate doses of alcohol at social occasions (social drinkers; Gonzales 2018). The experiment was approved by the ethics committee of the Medical Department of the University Erlangen‐Nuremberg. Prior to the testing, all participants received detailed information about the study protocol and provided written informed consent. After testing, all participants were fully debriefed and received monetary compensation for their participation.

**TABLE 1:** Sample characteristics (41 participants, 22 female) as well as breath alcohol concentration and pain threshold values.

	Mean	SD
Age (years) Range: 30–60 years	44.78	9.44
Weight (kg) Range: 52–114 kg	76.14	14.32
AUDIT Range: 0–9	4.02	2.20
BDI Range: 0–10	4.00	2.71

All participants (*N* = 41) underwent all three conditions		Placebo		Low dose		High dose	
		Mean	SD	Mean	SD	Mean	SD
BrAc (‰)	1	0.00	0.00	0.52	0.15	0.67	0.21
	2	0.00	0.00	0.40	0.13	0.51	0.16
	3 (before starting the post testing block)	0.00	0.00	0.53	0.11	0.72	0.12
	4	0.00	0.00	0.42	0.11	0.58	0.10
Pain thresholds (°C)	Pre testing block	45.26	1.16	45.02	1.13	45.14	1.12
	Post testing block	45.10	1.29	45.20	0.95	45.23	1.13

Abbreviations: AUDIT, Alcohol Use Disorders Identification Test; BDI, beck depression inventory; BrAc, breath alcohol concentration; SD, standard deviation.

### 2.2 Procedure

**FIGURE 1:**
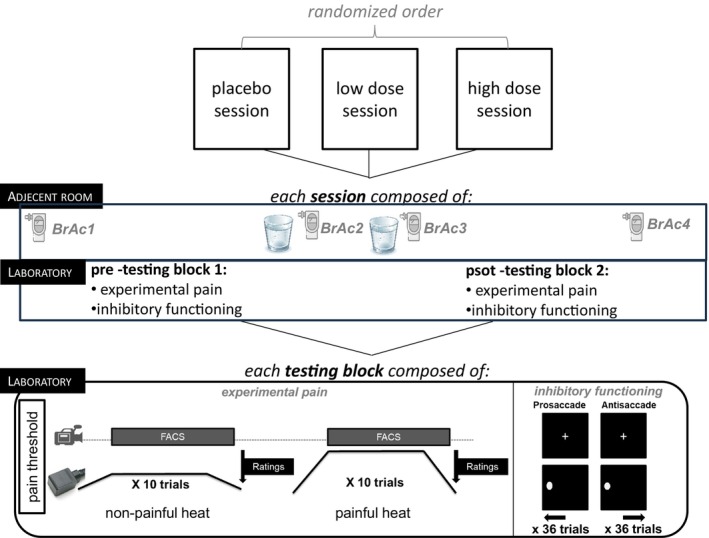
Study design of the present study.

Participants took part in three sessions (see Figure 1). Each session lasted for about 2.5 h and consisted of two testing blocks conducted before (pre testing block; T1) and after (post testing block; T2) drink administration (see Figure 1). Each testing block was composed of an experimental pain part and an inhibitory functioning part (see Figure 1). Breath Alcohol Content (BrAc) measurement was assessed at four time points throughout each session. At the end of each experimental session, participants' beliefs regarding the nature of the condition (alcohol or placebo) were assessed prior to the final BrAc measurement (Do you think you have received alcohol in this session?). In order to assure blinding of the experimenter, the drink administration was conducted by a research assistant in a second room adjacent to the laboratory (see Figure 1).

The order of drinking conditions (placebo, low dose, high dose); with one condition per session was randomised across participants. Test sessions were separated by 1–6 days and took place in the afternoon in the experimental laboratory of the Department of Physiological Psychology at the University of Bamberg.

### 2.3 Drink Administration and BrAc Measurements

#### 2.3.1 Beverage

A non‐alcoholic cocktail consisting of lime juice (20 mL), blue curacao syrup (40 mL) and bitter lemon (120 mL for the placebo drink, 120 mL minus the individually calculated amount of alcohol for the alcoholic drink) served as the basis for alcohol and placebo drinks. The intense bittersweet taste of this cocktail was chosen to complicate the evaluation of whether the drink contained any alcohol. Ethyl alcohol (70% vol.) was added to the cocktail in the alcohol drinking conditions and was sprayed onto the rim of the glass containing the placebo drink to mimic the alcoholic scent. Thus, there was a smell of alcohol in each condition (alcohol as well as placebo); the spraying of the rim in the placebo condition did, however, not change the BrAc. Participants received 2 glasses (180 mL each) of the cocktail successively. Each glass had to be consumed within 5 min. Ten minutes after consumption of each glass, BrAc measurements were taken (Scheel et al. 2013; Swift 2003).

#### 2.3.2 BrAc Measurements

Participants breath alcohol contents were measured regardless of alcohol or placebo drinking condition using a standard handheld breathalyser (DRÄGER Alcotest 7410Plus, Dräger Medical GmbH, Lübeck): BrAc (1) at the beginning of each session to assure that participants were sober (BrAc = 0.0‰) upon arrival; BrAc (2) 10 min after the consumption of the first glass; BrAc (3) 10 min after the consumption of the second glass, and BrAc (4) at the end of each session (see Figure 1). Participants were informed of the result of the BrAc 4 measurement; if it was above 0.3‰, we recommended participants stay in the hallway outside adjacent to our laboratory. In this case, we re‐assessed BrAc measurements every 20 min until this target value was reached and the participant was sent home.

#### 2.3.3 Titration of Doses

We adjusted participants' BrAcs to targeted values of 0.6‰ (low dose) and 0.8‰ (high dose). Participants received two glasses (180 mL each) of the cocktail successively (see Figure 1). The BrAc 2 (after the first glass) was used to adjust the amount of alcohol to be added in the second glass. The amount of alcohol per subject and condition was calculated by using the Widmark‐Formula (Widmark 1932). Participants drank the first 200 mL‐glass containing the cocktail either without alcohol or mixed with 75% of the calculated alcohol dose. Afterwards, bridging the period of alcohol absorption, participants played a computer game (participants had to indicate as fast as possible the colour of a circle appearing on the middle of the computer screen). Then, they completed BrAc 2 and received a second cocktail. In the alcohol drinking conditions, a second alcohol dose was calculated again using the Widmark‐Formula (by taking into account the difference between reached BrAc 2 and the target value of 0.6‰ or 0.8‰). In case of BrAc 2 values < 75% of the target value, the difference to the target value was added to these 25% to prevent large variation regarding BrAcs, which might be caused by interindividual differences in alcohol metabolism. Again, participants played the computer game and completed BrAc 3.

### 2.4 Testing Blocks

#### 2.4.1 Experimental Pain Induction

Pain testing was conducted twice in each session, before (pre testing block) and after (post testing block) drink consumption and consisted of the determination of pain threshold and a phasic heat stimulation paradigm where brief contact heat stimuli were applied and subjective ratings as well as the facial expression were recorded (see Figure 1). Pain was induced experimentally by a Peltier‐based contact stimulation device (Medoc TSA‐2001; Medoc, Ramat Yishai, Israel) according to a protocol that has successfully been used in our lab (Kunz et al. 2011). A 30 × 30 mm^2^ contact thermode was attached on three designated sites on the volar site of the left forearm. Temperature intensities were tailored to the individual heat pain threshold. This was done in order to ensure that participants perceived the pre‐set stimuli as similarly painful and to prevent floor as well as ceiling effects.

##### 2.4.1.1 Pain Threshold Determination

Heat pain thresholds were determined twice in each session (pre and post testing blocks) using the method of adjustment. The thermode was attached medially on the volar side of the left forearm. Participants were asked to adjust a temperature starting from 38°C that they perceived as barely painful by pressing heating and cooling buttons (rate of change 0.5°C/s by constant pressing). Following a familiarisation trial, there were four trials, and the average of these trials served to establish the threshold estimate.

##### 2.4.1.2 Phasic Heat Stimulation

Phasic heat stimuli were applied to the volar side of the left forearm. To avoid local skin sensitisation, probe position was slightly changed upwards (pre testing block) and downwards (post testing block) after the determination of pain threshold. Each stimulus had the same characteristics (trapezoidal shape; 5 s plateau duration; rate of change 4°C/s; baseline temperature 35°C; inter‐stimulus intervals of 15–20s). Each participant received ten non‐painful heat stimuli (pain threshold −1°C) and ten painful stimuli (pain threshold +3°C) in a pseudo‐random order (see Figure 1). Stimulus intensities were always based on the preceding pain threshold assessment (pre testing block [T1 threshold −/+ 1°/3°C], post testing block [T2 threshold −/+ 1°/3°C]).

#### 2.4.2 Pain Responses

##### 2.4.2.1 Pain Ratings

Participants rated pain intensity (the sensory pain dimension) and pain unpleasantness (the affective pain dimension) of the stimuli on two eleven‐point Numerical Rating Scales (NRS), ranging from 0 (“not painful/unpleasant”) to 10 (“extremely painful/unpleasant”) (Price et al. 1983). Both scales appeared consecutively on the computer screen after stimulus offset. Participants provided ratings by mouse click. Ratings were averaged across the 10 non‐painful and the 10 painful stimuli, and these mean values were used for further analyses.

##### 2.4.2.2 Facial Expression

In order to analyse facial expressions during heat stimulation, the face of the participant was videotaped throughout the pain testing block. The camera was placed in front of the subject at a distance of approximately 2 m. Participants were instructed not to talk during thermal stimulation and to always focus on the computer screen to await the appearance of the rating scales. A light‐emitting diode visible to the camera, but not to the participant, was lighted concurrently with the thermal stimuli to mark the onset of stimulation (see Figure 3). A software designed for the analysis of observational data (The Observer XT; Noldus Information Technology, Wageningen, Netherlands) was used to segment the videos and to enter the facial expression codes into a time‐related database. Time epochs of 7 s—beginning just after the stimulus had reached the target temperature (including plateau and ramp down)—were analysed off‐line. For each of the three experimental sessions, 40 trials of thermal stimulation were analysed in each subject ([10 non‐painful +10 painful trials] × two blocks [pre and post testing blocks]). All coding of facial expression was blind regarding the session (placebo, low dose, high dose) and the block (pre and post testing blocks). Facial responses were quantified using the Facial Action Coding System (FACS; Ekman and Friesen 1978), a fine‐grained anatomically based system that is considered the gold standard for analysing the facial expression of pain (Kunz et al. 2019; Craig et al. 2001). The intensity (5‐point scale) and frequency of facial Action Units (AUs) were rated off‐line by two certified FACS coders. Interrater reliability was calculated based on 20% of the video recording using the Ekman–Friesen formula (Ekman and Friesen 1978) and reached 0.84, which compares favourably with other research in the FACS literature. To select those AUs that were relevant to pain in the present experimental context, we used the following steps: (i) AUs had to occur in more than 5% of the painful segments recorded and (ii) AUs had to be more frequent during pain than during non‐painful stimulation (effect sizes *d* ≥ 0.5). Only those AUs that proved to be pain‐indicative in all sessions and in each testing block were selected for further analyses (these AUs are marked with a * in Table 2). In order to generate a measurement for the overall facial expression of pain, we formed pain‐indicative composite scores out of the selected AUs (AUs 4, 6_7, 9_10, 25_26_27; see Table 2) by first multiplying mean intensity and frequency values separately for each AU and then averaging the values to form a composite score.

**TABLE 2:** Percentage of occurrence (%) of facial Action Units (AUs) during painful segments are displayed. Moreover, effect sizes (Cohens *d*) are listed for the differences in occurrence between painful and non‐painful heat stimulation. Effect sizes *d* > 0.5 (moderate effect size) are marked in bold.

Action units		Placebo session				Low dose session				High dose session			
		pre		post		pre		post		pre		post	
		%	*d*	%	*d*	%	*d*	%	*d*	%	*d*	%	*d*
AU 1_2	Raised eyebrows	9	0.29	8	0.16	19	**0.52**	14	**0.55**	9	0.43	16	0.48
AU 4*	Furrowed brows	20	**0.85**	22	**0.81**	29	**0.85**	27	**1.14**	21	**1.05**	30	**1.19**
AU 6_7*	Narrowed eyes	95	**1.24**	86	**1.52**	100	**1.39**	101	**1.50**	81	**1.33**	95	**1.13**
AU 9_10*	Raising upper lip	24	**0.75**	17	**0.50**	27	**0.64**	31	**0.62**	21	**0.74**	32	**0.82**
AU 14	Dimpler	19	**0.70**	17	0.32	25	**0.80**	28	**0.59**	12	0.24	16	0.28
AU 17	Chin raiser	8	—	—	—	5	—	—	—	8	—	6	—
AU 18	Lip pucker	10	**0.55**	6	0.10	7	0.41	8	0.16	7	0.26	8	**0.53**
AU 23	Tightened lips	6	—	7		—	—	6	—	—	—	—	—
AU 25_26_27*	Opened mouth	24	**0.65**	23	**0.72**	20	**0.67**	29	**0.68**	22	**0.76**	28	**0.56**
AU 43	Closed eyes	6	—	6	—	5	—	9	—	—	—	8	

* Action Units selected for further analyses because they occurred in > 5% of all painful segments and occurred more frequently during painful compared to non‐painful segments (effect size > 0.5) in all conditions.

#### 2.4.3 Inhibitory Functioning

##### 2.4.3.1 The Antisaccade Task

The antisaccade task was always conducted following the experimental pain testing and was based on established protocols (Derakshan et al. 2009; Karmann et al. 2015). Stimulus presentation and tracking of eye movements were conducted using the system of Interactive Minds (Dresden, Germany). This system consisted of a 19 in. Samsung LCD screen (resolution 1280 × 1024 pixels; 60 cm viewing distance) and the eyetracking system Eyegaze Edge by LC Technologies Inc. and was driven by the software NYAN 2XT (version 2.3.3). In order to measure the pupil's orientation, this eyetracking system uses the corneal reflection of an infrared light source (corneal reflex method; Mason 1969). It features a sampling rate of 60 Hz and a fidelity of 0.4°.

Each trial began with the presentation of a fixation cross (12 mm × 12 mm, 1.15° × 1.15°) in the centre of the screen which was to be fixated until it disappeared (2000 ms) (see Figure 1). Concurrently, two dark grey rectangular frames were presented left and right of the fixation cross (60 mm × 60 mm, 5.73° × 5.73°; located at 8.29° horizontally from the fixation cross) to indicate the destination of the later cue, on which the participants had to respond by a towards or an away saccade. These two frames were then constantly present in all following images. Next, a circle (diameter = 35 mm, 3.34°) which represented the cue appeared for 600 ms. The cue was either presented within the frame on the left or right side of the screen (11° horizontally from the fixation cross) with both locations being equally probable. Depending on the instruction given at the beginning of each block, participants were asked to look either towards the cue (prosaccade block) or away from it (antisaccade block), as quickly as possible. Afterwards, an arrow pointing up or down (11 mm × 24 mm, 1.05° × 2.29°), which served as the target stimulus, was presented for 100 ms. The target either appeared in the same location as the cue (prosaccade block) or on the opposite side of the screen (antisaccade block). The participants had to identify the direction of the arrow by pressing the up or down key of a regular computer keyboard as fast and accurately as possible. Since eye movements were the parameter of interest indicating motor inhibition, key‐press reactions were not further analysed. The complete antisaccade task consisted of six blocks (three prosaccade and three antisaccade blocks; alternating), with 12 trials each, which resulted in a total of 72 trials. One half of the participants started the test with a prosaccade block, the other half with an antisaccade block. Ahead of testing, participants practiced the task in a short training block consisting of eight trials.

The measures chosen for further analyses were—based on previous work (Derakshan et al. 2009)—the following: Latency of the first correct saccade (latency of the first saccade (time interval between cue onset and start of the first fixation) in the right direction) and percentage of incorrect saccades (percentage of first saccades in the wrong direction). Inhibitory functioning was then determined by calculating the differences between anti‐ and prosaccade trials for the two variables (in the following called ∆). High scores thus indicated a poor ability to inhibit (low degree of inhibitory functioning), whereas low scores indicated a high degree of inhibitory functioning.

### 2.5 Questionnaires

At the beginning of the first session, participants were screened for problematic alcohol misuse and depression using the German versions of the Alcohol Use Disorders Identification Test (AUDIT; Allen et al. 2001; Dybek et al. 2006) and the Beck Depression Inventory (BDI; Beck et al. 1961; Hautzinger et al. 2002). With an AUDIT cut‐off score of 9 or above (indicating possible risky drink behaviour) participants were additionally screened for alcohol abuse and addiction by use of the Structured Clinical Interview for DSM Disorders (SCID, First et al. 1995) and, in case of answers still indicating alcohol abuse, they were excluded from participation. Scoring the BDI above 19 (indicating possible moderate to severe depression), participants were excluded from participation. None of the participants had to be excluded after screening for alcohol misuse and depression.

### 2.6 Statistical Analysis

As manipulation check, the administered alcohol doses and BrACs measured after the second glass (BrAC 3) were compared between the two alcohol conditions using dependent samples *T*‐tests. In addition, subjective beliefs concerning the nature of the condition (alcohol or placebo) were descriptively compared between conditions.

For the effect of alcohol on pain outcomes, we focused on facial and subjective responses to the painful heat intensities. Only in case of significant findings, the responses to the non‐painful heat were considered in order to test for pain specificity of the findings. Alcohol effects on the outcome measures (pain ratings, facial responses to pain, antisaccade task) were evaluated by using analyses of variance with repeated measurements with the following within‐subject factors: ‘condition’ (placebo, low dose, high dose) × ‘pre‐post’ (pre and post testing blocks). In case of pain ratings and the antisaccade task, we used multivariate analyses of variance to account for the two parameters of each of the dependent variables (ratings: intensity & unpleasantness ratings; antisaccade task: reaction time & percentage of errors). Post hoc *T*‐tests were computed for detailed analysis.

All findings were considered to be significant at *α* ≤ 0.05. For *F*‐tests, partial eta squared (*ƞ* ^2^) as an estimate of effect size is reported (0.01: small effect; 0.06: medium effect; 0.14: large effect) (Pierce et al. 2004). For paired comparisons, we report Cohen's *d* (0.20: small effect; 0.50: medium effect; 0.80: large effect) (Cohen 1988). Data was analysed using SPSS (Statistical Package for the Social Sciences, IBM, Armonk, NY, USA) version 29 for Windows.

Part of the dataset assessed in this study (pain thresholds and pain ratings) has already been published (Capito et al. 2020), due to other hypotheses, the statistical analyses and outcomes from Capito et al. (2020) differ from the current ones.

## 3 Results

### 3.1 Descriptive Sample Characteristics

AUDIT scores (see Table 1) were below the cut‐off of 9 in all 39 participants; two participants reached a score of 9, but did not report any DSM V diagnostic criteria of alcohol use disorders. Likewise, BDI scores (see Table 1) were within the norm values for non‐clinical samples (Hautzinger et al. 2002; Beck et al. 1961).

### 3.2 Manipulation Check

Mean breath alcohol levels after the second glass (BrAc 3) were close to the respective target values for both alcohol conditions (see Table 1). As expected, BrAc 3 was significantly higher in the high dose compared to the low dose sessions (*T* [40] = 11.170, *p* < 0.001). In addition, the mean administered alcohol doses in total (low dose: *M* = 66.48 ± 18.88 g; high dose: *M* = 89.92 ± 26.76 g) and in relation to participants' body weight (low dose: *M* = 0.49 ± 0.08 g/kg; high dose: *M* = 0.66 ± 0.12 g/kg) were in the expected range and significantly higher in the high dose compared to the low dose condition (Dose_total_: *T* (40) = 10.319, *d* = 0.88; Dose_g/kg_: *T* (40) = 12.166; both *p*‐values < 0.001). These results corroborated the successful implementation of two different alcohol conditions. In the placebo condition, 59% (*N* = 24) of the participants believed that they had received an alcohol drink, thus proving the successful induction of uncertainty regarding the nature of this drink. In contrast, almost all participants were sure that they had received alcohol in both alcohol conditions (low dose: 95%, *N* = 39; high dose: 93%, *N* = 38). There was no difference in the pain parameters and the antisaccade task between those (59%) who believed to have received alcohol in the placebo session and those (41%) who believed to have not (*F* [4, 35] = 0.42, *p* = 0.791).

### 3.3 Alcohol Effects on Pain Outcomes

Given that pain intensities to evoke facial responses were always tailored to the individuals pain threshold, we first checked whether alcohol consumption had an effect on pain thresholds. Comparing pain thresholds between pre and post testing blocks (separately for all sessions) did not show any significant changes (all *p*‐values > 0.05). Pain threshold values are given in Table 1.

#### 3.3.1 Pain Ratings

**FIGURE 2:**
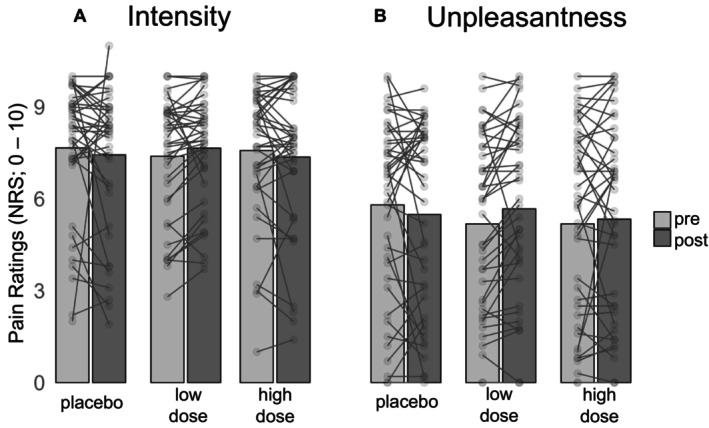
Descriptive statistics (Mean, individual data points) of (A) pain intensity and (B) pain unpleasantness NRS ratings of painful heat stimulation assessed before (pre) and after (post) drink administration in each of the three drink conditions.

Multivariate (pain intensity and unpleasantness ratings) analysis of variance with repeated measurement showed no significant main effects; neither for the factor ‘condition’ (*F* [4, 160] = 0.503; *p* = 0.734; *ƞ* ^2^ = 0.01) nor for the factor ‘pre‐post’ (*F* [2, 39] = 1.959; *p* = 0.155; *ƞ* ^2^ = 0.09). Moreover, the interaction between the two factors was also not significant (*F* [4, 160] = 1.639; *p* = 0.167, *ƞ* ^2^ = 0.04). As can be seen in Figure 2, pain ratings (both for pain intensity and pain unpleasantness) remained relatively stable across sessions as well as across pre and post testing blocks.

#### 3.3.2 Facial Expression of Pain

**FIGURE 3:**
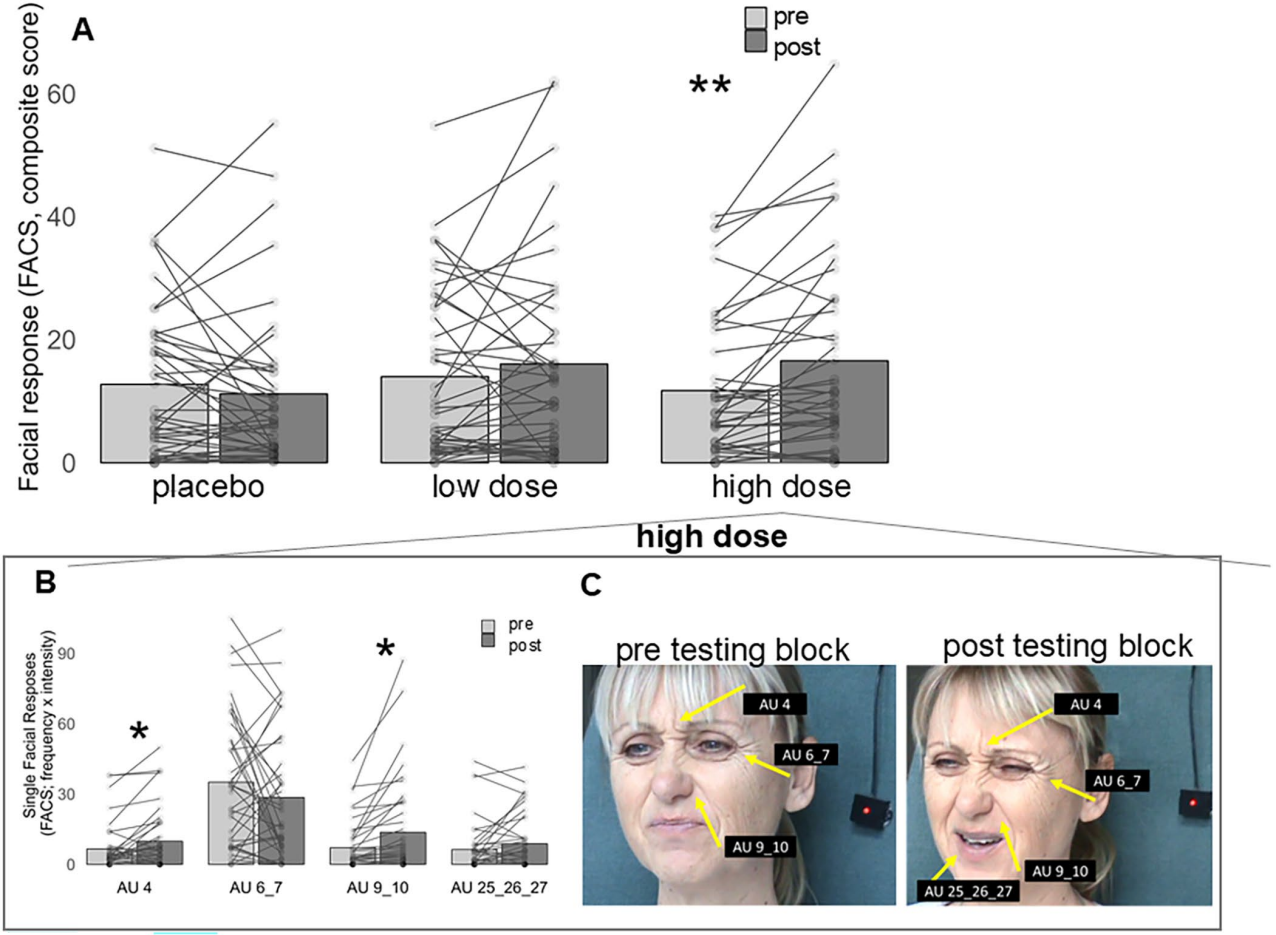
Descriptive statistics (Mean, individual data points) of facial responses to painful heat stimulation assessed before (pre) and after (post) drink administration. Results are presented (A) for the composite score of facial responses and (B) for the selected pain‐indicative single Action Units during the higher alcohol condition. (C) Shows an example of facial expression of a participants pre‐ and post‐alcohol consumption in the higher alcohol condition. Asterisks indicate significant differences (*p < 0.05, **p < 0.01).

Analysis of variance with repeated measurement yielded no significant main effects; neither for the factor ‘condition’ (*F* [2, 80] = 2.254; *p* = 0.112, *ƞ* ^2^ = 0.05) nor for the factor ‘pre‐post’ (*F* [1, 40] = 3.544; *p* = 0.070, *ƞ* ^2^ = 0.08). However, the interaction between these two factors yielded a significant interaction effect (*F* [2, 80] = 3.714; *p* = 0.029, *ƞ* ^2^ = 0.09). As can be seen in Figure 3A, facial responses to pain significantly increased from pre to post testing blocks only when participants consumed the higher dose of alcohol whereas no significant changes were found for the placebo condition or for the low alcohol dose. Thus, the higher dose of alcohol led to an increase in facial responses to pain.

To better understand whether the higher dose of alcohol led to an increase in all of the selected pain‐relevant AUs (see Table 2 for selection of AUs and Figure 3B for examples), we computed further analyses. Separately for each of the four AUs, paired comparisons (dependent sample *t*‐tests (one‐tailed), Bonferroni correction with four comparisons) between pre and post testing blocks were computed for the high dose condition. This analysis yielded significant increases in facial activity for AU 4 (furrowed brows) and AU 9_10 (raising upper lip) whereas no significant increase was found for AU6_7 (narrowed eyes) and AU25_26_27 (opened mouth) (see Figure 3B). Thus, the significant increase in facial responses to pain due to the higher dose of alcohol (see Figure 3A) was mainly due to an increase in AU 4 and AU 9_10 (see Figure 3B).

Given the significant effects of alcohol on facial responses to painful heat, we also computed an analysis of variance with repeated measurement for facial responses during non‐painful heat stimulation. For the non‐painful heat intensity, no significant main effects (‘condition’) (*F* [2, 80] = 0.191; *p* = 0.182 ƞ2 = 0.01); ‘pre‐post’ (*F* [1, 40] = 0.363; *p* = 0.550; *ƞ* ^2^ = 0.01) or interaction effect (*F* [2, 80] = 0.169; *p* = 0.845, ƞ2 < 0.01) were found.

### 3.4 Alcohol Effects on Inhibitory Functioning

**FIGURE 4:**
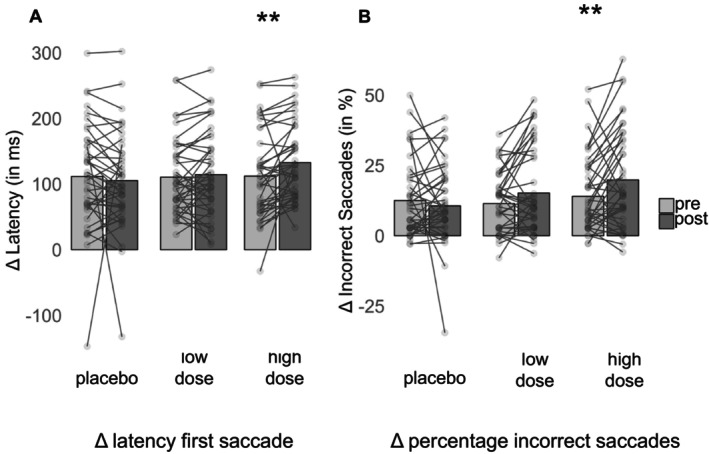
Descriptive statistics (Mean, individual data point) of the antisaccade task assessed before (pre) and after (post) drink administration in each of the three drink conditions. Asterisks indicate significant differences (*p < 0.05, **p < 0.01).

Multivariate analyses (∆latency of the first correct saccade, ∆ percentage of incorrect saccades) of variance with repeated measurement yielded no significant main effect for the factor ‘pre‐post’ (*F* [2, 39] = 1.874; *p* = 0.167, *ƞ* ^2^ = 0.09). However, there was a main effect for the factor ‘condition’ (*F* [4, 160] = 4.188; *p* = 0.003, *ƞ* ^2^ = 0.10), and a significant interaction effect between ‘condition’ and ‘pre‐post’ (*F* [4, 160] = 3.254; *p* = 0.013; *ƞ* ^2^ = 0.08), with inhibitory functioning decreasing after alcohol intake (see Figure 4). As post hoc *T*‐tests showed, ∆latency of the first correct saccade significantly increased after participants consumed the higher dose of alcohol (*p* = 0.003) whereas no significant changes were found for the placebo condition (*p* = 0.553) or for the lower alcohol dose (*p* = 0.445) (see Figure 4). With regard to the ∆percentage of incorrect saccades, post hoc *T*‐tests showed a significantly increased percentage of incorrect saccades after alcohol intake (low dose: *p* = 0.031; high dose: *p* = 0.009) whereas no significant changes were found for the placebo condition (*p* = 0.429) (see Figure 4). Thus, especially the higher dose of alcohol led to a decrease in inhibitory functioning.

### 3.5 Association Between Alcohol‐Induced Changes in Facial Responses to Pain and Inhibitory Functioning

**FIGURE 5:**
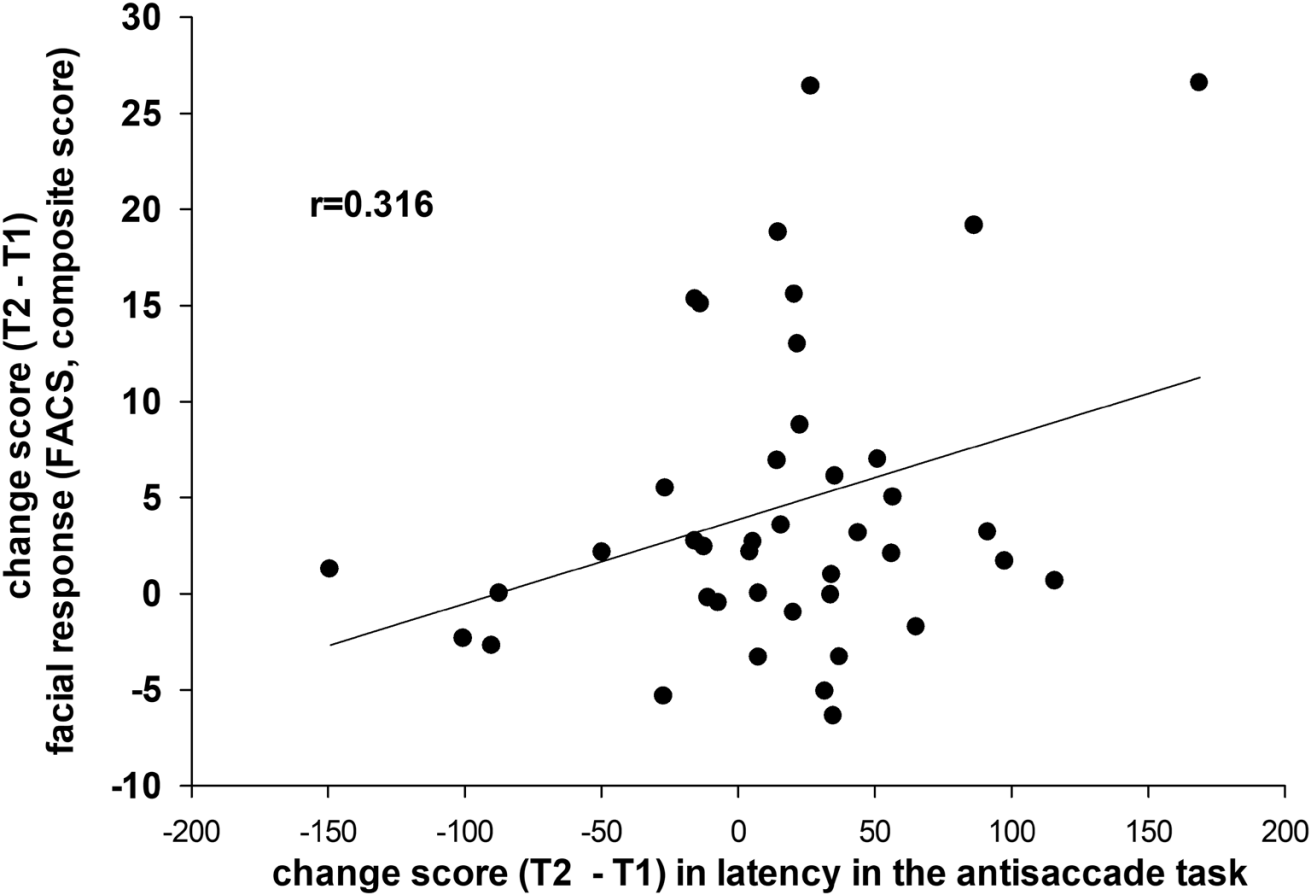
Association between alcohol‐induced changes (post–pre testing blocks) in (i) facial responses and (ii) in the latency response of the antisaccade task during the higher alcohol condition.

As a final step, we wanted to test whether the alcohol‐induced changes in facial responses and in inhibitory functioning are associated, as theoretically proposed in the introduction. Pearson correlation showed a significant association (*r* = 0.316, *p* = 0.044) between alcohol‐induced increase in facial responses and in the latency of the first saccade (see Figure 5). Thus, the greater the decline in inhibitory functioning (latency of the response) the greater the disinhibition in facial responses to pain. Changes in the percentage of incorrect saccades were not associated with changes in facial responses (Pearson correlation, *r* = −0.128; *p* = 0.423).

## 4 Discussion

The main finding of the present study was an increase in facial responses to pain despite almost unchanged psychophysical pain measures following the oral consumption of subtoxic doses of alcohol. This was mainly true for the highest dosage (0.72‰ BrAC on average). In other words, an alcohol‐induced elevated facial display of pain was accompanied by an unaffected subjective pain experience. Notably, heat pain stimuli around 48°C and NRS ratings for pain intensity of approximately 8 out of 10 confirmed that experimental pain was induced at a moderate to high level, allowing for the demonstration of analgesic effects. Given that similar studies demonstrated weak analgesic effects of comparable alcohol doses in psychophysical paradigms (Thompson et al. 2017), facial responses to pain—showing effects in the opposite direction—do not appear suitable for experimentally demonstrating alcohol‐induced analgesia.

The clear increase in the facial display of pain after the higher dose of alcohol rather suggests that a disinhibition of the facial response to pain occurred. This assumption is corroborated by our finding that the change in facial activity during pain from pre‐ to post‐alcohol consumption is significantly correlated with the change in our motor inhibition test, namely the antisaccade task after consumption of the higher alcohol dosage. Thus, those participants with an alcohol‐induced stronger facial response to pain are also characterised by poorer antisaccade performance, that is, low motor inhibition after alcohol intake. In essence, alcohol in sub‐toxic doses leads to a disinhibition of the facial display of pain. This effect depends on the modulation of pain‐indicative contractions of facial muscles, which are controlled via a final motor pathway not involved in the generation of psychophysical pain ratings. This separation may explain the weak correlations often observed between facial expressions of pain and subjective pain reports (Kunz et al. 2004).

These findings further strengthen our model of how facial activity during pain is regulated. Facial responsiveness to pain is negatively correlated with inhibitory mechanisms regulating motor responses, which are often implemented in prefrontal regions (Karmann et al. 2015). Correspondingly, previous work conducted in the lab found prefrontal areas being involved in regulating facial responses to pain (Kunz et al. 2011, 2023). In accord, we could manipulate the facial display of pain by deactivation of these areas, using repetitive transcranial magnetic stimulation (Karmann et al. 2016). Thus, facial responses to pain are not only generated by activation of typical nociceptive brain areas (S1, insula, anterior cingulate cortex) but are also controlled by very efficient motor output gates (prefrontal areas), regulating the response channels for the facial display of pain. We have now demonstrated that these prefrontal gates are sensitive to alcohol, which is a substance known to trigger motor disinhibition (Miller et al. 2012; Rose and Duka 2007). The inhibitory motor gates in the prefrontal cortex, which control the degree of facial expressiveness, were apparently opened (disinhibited) by the highest alcohol dosage only.

Interestingly, certain Action Units, which have shown closer linkage to the affective dimension of pain, that is, furrowed brows (AU 4) and raising of the upper lip (AU9_10) (Kunz et al. 2012; Blais et al. 2019), seem to be more sensitive to this disinhibitory action of alcohol. Thus, it may well be that the motor output control of the facial pain response consists of several channels, which allow for the production of different facial patterns of facial responses to pain; in contrast to the idea of one generally acting motor gate for the face, which only allows for all or nothing actions. Following this line of argument, alcohol might specifically disinhibit the facial display of pain unpleasantness or the affective dimension of pain, whereas the facial encoding of pain intensity (AU 6_7) (Kunz et al. 2020) was less affected by alcohol. Thus, we assume by speculation at least two prefrontal gates: one responsible for the facial encoding of the affective dimension of pain, which can be opened by alcohol (and likely other sedative or anxiolytic agents), and another regulating the encoding of the sensory dimension, which appears to be unaffected by such substances. These findings are also in line with a review article that we conducted on the effect of alcohol on facial expressions of emotions (Capito et al. 2017). Across studies, the effect of alcohol on facial expressions is apparently not due to a general disinhibition of all facial responses, but the facial effects varied depending on the valence of emotion and on the type of social interaction.

Our findings have significant clinical implications. Consumption of alcohol elevates the risk of accidents and physical violence, which in turn can lead to injuries and hereby to acute pain. Under the influence of alcohol, people injured and in pain may subjectively perceive and rate their pain as being less intense due to the analgesic action of alcohol but may nevertheless facially display pain more strongly. Thus, the divergence between these two indications of pain (lower subjective vs. higher facial responses) may not reflect an intentional exaggeration by a slightly analgized person, but rather an accurate dual representation of pain under the influence of alcohol. In essence, the pain communication appears to be complicated by alcohol—with stronger facial signals and weaker verbal signals of pain—potentially leading to confusion for those diagnosing and treating pain. As a further consequence, enhanced facial responses to pain may serve as go‐ or stop‐signals during physical altercations, potentially contributing to the management of violence and aggression (MVA). Furthermore, the alcohol‐induced increase in facial responses to pain observed in the present study mirrors previous findings we reported in individuals who were either in the presence of their partner (Karmann et al. 2014) or had undergone induction of an optimistic state (Basten‐Günther et al. 2021). Thus, drinking up the courage may exert similar influences on the facial display of pain to other variables increasing daring attitudes likely associated with reduced anxiety levels.

We do not see major contradictions between the absence of significant effects of subtoxic doses of alcohol on verbal pain reports (e.g., NRS) in the present study and the positive findings reported in previous studies from our own and other labs (Capito et al. 2020; Horn‐Hofmann et al. 2015, 2019; Perrino Jr. et al. 2008; Thompson et al. 2017). There is general agreement that the analgesic effect of such dosages is weak, making it variably observable in small samples. One must keep in mind that the investigation of higher dosages of alcohol with toxic effects is not preferential because of the enhancement of a general behavioural disorganisation.

### 4.1 Limitation

The present study requires an enormous effort (e.g., three long sessions including time for recovery to soberness with intervals of several days, time‐consuming FACS coding of the facial pain responses), which makes increasing the number of participants a challenge and a potential risk to the feasibility of the project. Nevertheless, the observed effects would benefit from greater statistical power achieved through a larger sample size. Recently, we combined several of our studies on sex differences in the facial display of pain, which allowed us to identify clearer effects that had previously been inconsistent in individual studies (Schneider et al. 2024). This is merely an example of how power‐sensitive FACS‐coded data can be, and not a suggestion of a different topical focus.

## 5 Conclusion

We applied in a within‐subject placebo‐controlled design two subtoxic oral doses of alcohol, leading to BrAc levels of 0.53‰ and 0.72‰. The higher dose led to an increase in the facial display of pain, whereas subjective pain ratings were not affected. It seems reasonable to interpret this change in facial responses as alcohol‐induced motor disinhibition. Interestingly, just those facial Action Units indicating pain unpleasantness were sensitive to this alcohol action. These findings inform pain diagnosticians that enhanced facial expressiveness during pain in individuals under the influence of alcohol should not be interpreted as intentional exaggeration.

## Author Contributions

S.L. conceived and designed the study. C.H.‐H. and J.W. supervised the study. E.S.C. collected and organised the data. C.H.‐H. and M.K. performed data analysis. S.L., C.H.H., and M.K. drafted the manuscript. All authors discussed the results and commented on the manuscript.
